# Post elimination of lymphatic filariasis: a situation analysis of brugian filariasis and vector potentialities within the filarial transmission belt in Sri Lanka

**DOI:** 10.1186/s13071-026-07264-w

**Published:** 2026-03-06

**Authors:** Sachini U. Nimalrathna, Hiruni Harischandra, Nilmini Chandrasena, Michael J. Kimber, Nilanthi de Silva, Chandana H. Mallawarachchi, Thilina S. Nimalrathna, B. G. D. Nissanka K. de Silva

**Affiliations:** 1 Center for Biotechnology, Department of Zoology, Faculty of Applied Sciences, University of Sri Jayewardenepura, Nugegoda, Sri Lanka; 2 Department of Veterinary Pathology, Iowa State University, Ames, IA USA; 3 Genetics and Molecular Biology Unit, Faculty of Applied Sciences, University of Sri Jayewardenepura, Nugegoda, Sri Lanka; 4 Department of Parasitology, Faculty of Medicine, University of Kelaniya, Kelaniya, Sri Lanka; 5 Department of Biomedical Sciences, Iowa State University, Ames, IA USA; 6 School of Biological Sciences, University of Hong Kong SAR, Hong Kong, China

**Keywords:** Brugian filariasis vectors, Mosquito surveillance, *Brugia malayi*, Post-elimination, Sri Lanka

## Abstract

**Background:**

Sri Lanka is experiencing a re-emergence of brugian filariasis 4 decades after its elimination in 1969. A comprehensive understanding of the mosquito species that can facilitate the development of the brugian parasite is essential for implementing targeted surveillance and control measures. This study evaluated the vector potentiality of field-caught mosquitoes for brugian parasites across endemic districts within the filarial transmission belt in Sri Lanka.

**Methods:**

Mosquito surveillance was conducted at six sites across five districts with the highest reported brugian cases during 2021–2022. Mosquitoes were collected at the site of the most recently reported human brugian case in each district using dog-baited, window and gravid traps to maximize species diversity and abundance in the sample. Mosquitoes were identified morphologically, and randomly selected mosquitoes were molecularly confirmed via a PCR targeting the *COΙ* region. Vector potentiality was evaluated by observing nematode parasites upon dissection, molecular confirmation via PCR and sequencing the *Brugia sp.*-specific *HhaΙ* region. Mosquitoes harboring the infective L3 stage brugian parasites were tested for the presence of human DNA to investigate their involvement in human brugian filariasis transmission. Statistical analyses were performed using generalized linear mixed models.

**Results:**

A total of 766 mosquitoes of 15 species were dissected to obtain L3 larvae of the brugian parasite. Of these, 10.05% (*n* = 77) from nine species across four genera were identified to support the development of *Brugia* spp. to the infective L3 larval stage within the head and thoraces of field-caught mosquitoes: *Mansonia annulifera, Ma. indiana, Ma. uniformis*, *Culex lophoceraomyia, Cx. tritaeniorhynchus*, *Cx. quinquefasciatus*, *Cx. vishnui*, *Armigeres subalbatus* and *Coquillettidia crassipes.* Notably, *Ma. indiana*, which has not previously been identified as a potential vector for brugian filariasis in Sri Lanka, showed the highest weighted infectivity at the S_1_ site. Site-based risk assessment identified the S_1_ site as having the highest risk of brugian filariasis followed by S_6_.

**Conclusions:**

Many mosquito genera supporting the development of *Brugia* spp. to the infective L3 larval stage in field-caught mosquitoes were identified expanding beyond the previously known *Mansonia* vectors. The diversity of potentially infective species indicates complex transmission dynamics requiring integrated surveillance approaches.

**Supplementary Information:**

The online version contains supplementary material available at 10.1186/s13071-026-07264-w.

## Background

Lymphatic filariasis (LF) in humans is a mosquito-borne neglected tropical disease (NTD) caused primarily by *Wuchereria bancrofti*, *Brugia malayi* and *B. timori*. Globally, over 657 million people are threatened across 39 endemic countries, including Sri Lanka [1]. Commonly observed symptoms include hydrocele and lymphoedema, which can progress to elephantiasis, characterized by massive swelling of the extremities. These symptoms are attributed to the inflammatory reaction caused by adult parasites residing within the lymphatic vessels, leading to damage and dysfunction of lymphatic vessels and predisposing individuals to lymphoedema. This makes LF one of the leading causes of permanent disfigurement and the second leading cause of long-term disability [2, 3]. The severe morbidity of this disease poses a significant socioeconomic burden, hindering the development of affected communities in developing countries [4–8]. Consequently, the estimated financial burden exceeds $5.8 billion annually, covering treatment costs, healthcare costs and potential income losses [9].

In the past, both bancroftian filariasis and brugian filariasis (nocturnal periodic strain) were prevalent in Sri Lanka [10]. Before the LF elimination program, one-tenth of the inhabitants in the Sri Lanka's endemic regions were at risk of infection [11–14]. In the late 1960s, *B. malayi* was eliminated by removing the aquatic vegetation required for the known vector of the prevalent *B. malayi* strain to breed [10]. Following five successful mass drug administration (MDA) campaigns using diethylcarbamazine citrate (300 mg) combined with albendazole (400 mg) from 2002 to 2006, Sri Lanka received a certificate from the World Health Organization (WHO) in 2016 [15] for eliminating LF as a public health problem. However, post-elimination surveillance revealed the re-emergence of brugian filariasis, caused by what appears to be a variant of *B. malayi* [16–19].

This variant exhibits a nocturnal sub-periodic behavior, differing from the previously eliminated anthroponotic periodic strain [16, 20]. Molecular analysis based on the internal transcribed spacer region 2 (ITS2) suggests it may represent a novel genetic variant or hybrid strain of *B. malayi* and *Brugia pahangi* [17]. Alarmingly, surveillance reports indicate that brugian infections outnumbered bancroftian infections in 2023, highlighting the urgency of understanding transmission dynamics to prevent the re-establishment of endemic transmission [19].

Previous studies in Sri Lanka identified *Mansonia uniformis* and *Ma. annulifera* as competent vectors for the variant brugian strain [17], but a comprehensive assessment of the vector competence of the re-emerged variant is lacking, with only one study conducted in the Gampaha District, Sri Lanka [17]. Understanding vector competence for this variant is critical for several reasons; different parasite strains may have different vector competence patterns, post-elimination environmental changes may have altered vector populations, and effective vector surveillance and control strategies require knowledge of all competent vector species. This study aimed to assess the vector potentiality of mosquito species for brugian parasites across endemic districts in Sri Lanka, providing essential data for evidence-based surveillance and control strategies in the post-elimination era.

## Methods

### Study site selection

**Fig. 1:**
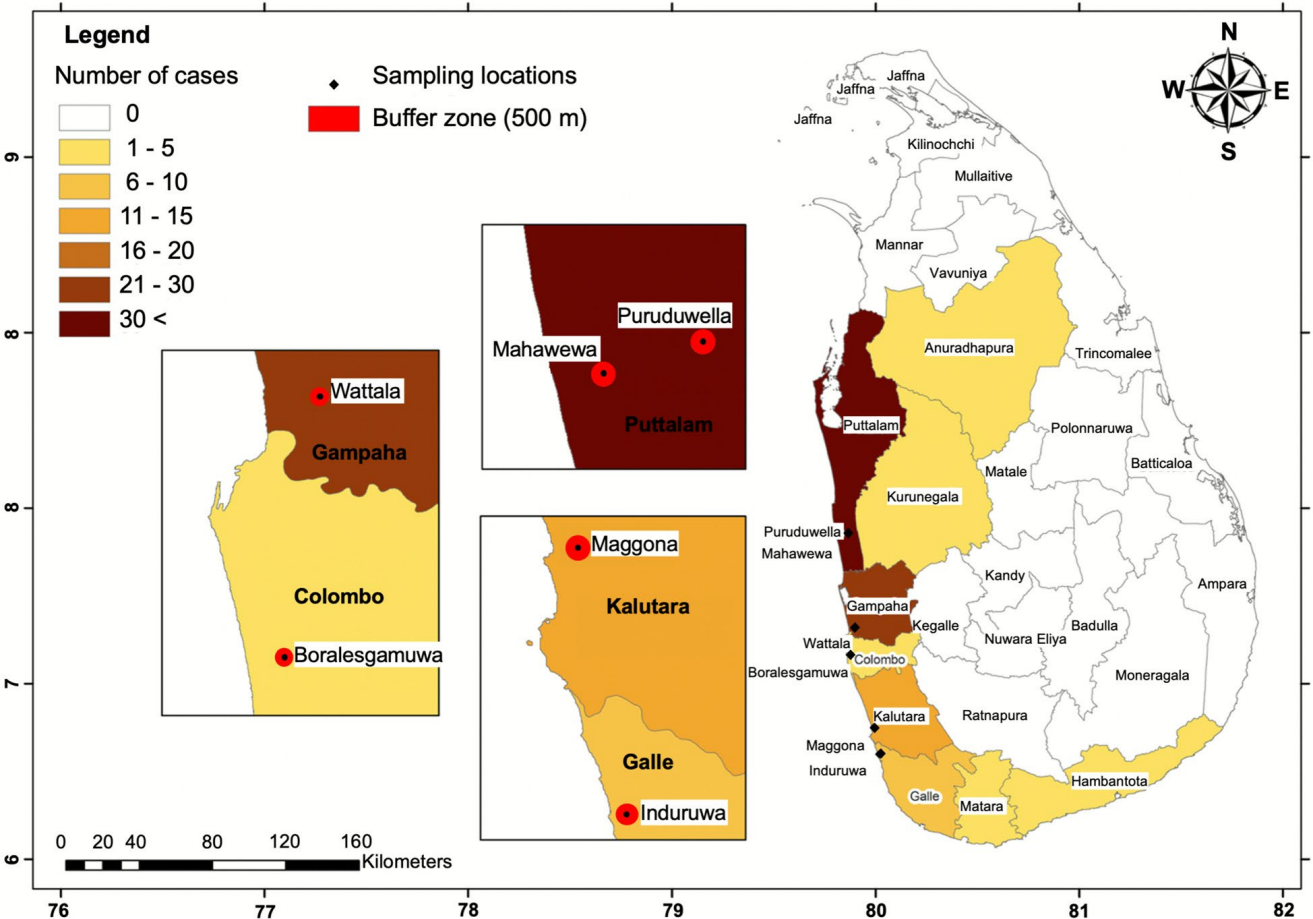
Distribution of brugian filariasis cases in Sri Lanka and mosquito collection sites. Districts in the filariasis belt include Puttalam, Gampaha, Colombo, Kalutara, Galle and Matara. Locations of recent positive cases from each district are shown as a black dot; Puruduwella-Puttalam District (S_1_), Maggona-Kalutara District (S_2_), Wattala-Gampaha District (S_3_), Induruwa-Galle District (S_4_), Boralesgamuwa-Colombo District (S_5_) and Mahawewa-Puttalam District (S_6_). Districts are colored based on the human cases reported between 2006 and April 2021

Five districts along Sri Lanka’s LF belt with the most cases of *Brugia* infection detected by the Anti-filariasis Campaign (AFC) via thick blood smear (TBS) observations (2002–2021) were selected: Puttalam (53 cases), Kalutara (32 cases), Gampaha (23 cases), Galle (10 cases) and Colombo (6 cases). The most recently reported human case of *Brugia* infection in each district at the time of the study (April 2022) was identified, and the index house was selected as the study site in each district. Two sites were chosen from the Puttalam District because of the high case incidence. The selected mosquito collection sites were Puruduwella-Puttalam District (S_1_: 7.4898707, 79.859083), Maggona-Kalutara District (S_2_: 6.51012, 79.992529), Wattala-Gampaha District (S_3_: 6.99634, 79.89580), Induruwa-Galle District (S_4:_ 6.3771134, 80.0171609), Boralesgamuwa-Colombo District (S_5_: 6.86579, 79.87821) and Mahawewa-Puttalam District (S_6_: 7.465026, 79.8697312) (Fig. 1).

### Mosquito collection

**Fig. 2:**
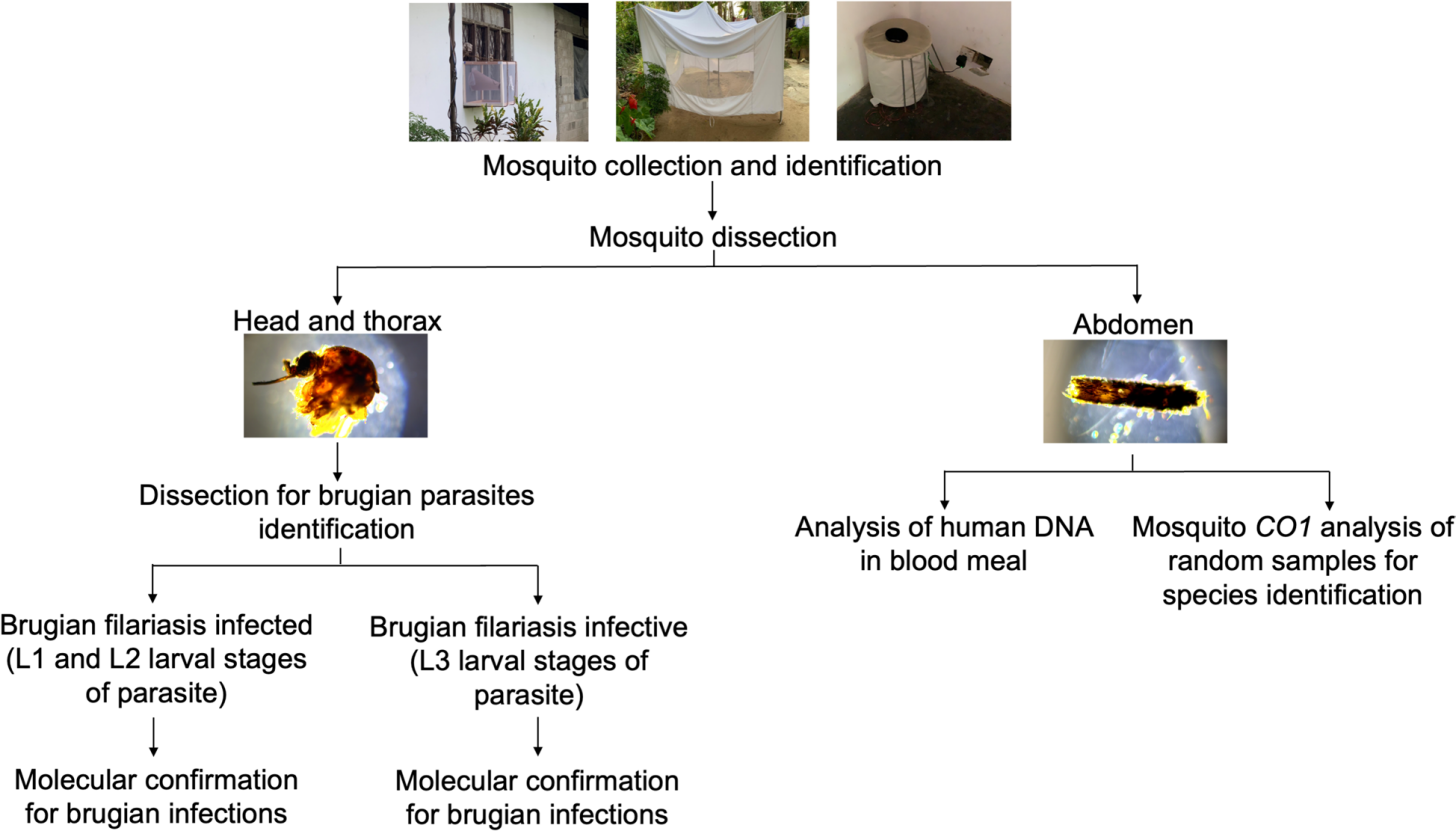
Generalized work flow of the study

A generalized workflow of the study is shown in Fig. 2. Mosquitoes were collected at S_1_ in October 2021, at S_2_ in December 2021, at S_3_ in February 2022, at S_4_ and S_5_ in June 2022 and at S_6_ in September 2022. Mosquitoes were collected using dog-baited traps, gravid traps and window traps to maximize species diversity and abundance in the mosquito samples. Mosquito collections were carried out over at least 2 consecutive nights at each location between 18:00 and 04:00 Sri Lankan Standard Time. Two of each trap type were installed at each study site to ensure consistency across study sites and to minimize sampling bias. For dog-baited traps, a dog was secured overnight within a white, rectangular, tent-like structure of nylon mesh (4 m × 3 m × 3 m), which was raised about 15 cm from the ground to allow mosquitoes to enter. The following morning, the dog was released, and the mosquitoes were collected using a mouth aspirator. Window traps measuring 56 cm × 56 cm × 46 cm [22] were placed outside the windows to collect anthropophilic mosquitoes exiting after a blood meal. The rest of the window was obstructed to ensure mosquitoes exited only through the opening leading to the trap. The trap was left in place overnight, and mosquitoes were collected using mouth aspirators the following morning. Gravid traps with attractants for *Culex* spp. were used to ensure their representation in the sample. An infusion of water and organic material (300 g fresh cow manure, 150 g *Gliricidia* leaves, 10 g yeast and 5 l water), fermented for 1–4 weeks, was placed in a 20 cm × 39 cm × 32 cm plastic dishpan with a motorized suction trap and left overnight. Mosquitoes were collected the following morning using a mouth aspirator and were transferred to holding containers. Although traps were left overnight for mosquito collection, all devices were designed to securely contain captured mosquitoes, preventing escape and thereby eliminating any risk of transmission from the dog-baited or window traps during the collection period. Mosquitoes were immobilized using chloroform to avoid physical damage, and head and thorax were dissected within 4 h to ensure detection of developing filarial larval stages within the mosquito tissues rather than microfilariae ingested during the most recent blood meal in the abdomen. The species of all the collected mosquitoes were identified using standard morphological keys [23, 24]. A subset of randomly selected mosquitoes was molecularly identified using the cytochrome c oxidase I *(COI)* region to validate the morphological identification.

### Detection of infected and infective mosquitoes

#### Mosquito dissection

Female mosquitoes were held for 4 h post-collection to allow microfilariae ingested with the most recent blood meal to exit the midgut into the hemocoel, after which only the head and thorax were dissected to detect brugian parasites developed to the infective L3 larval stage. Each mosquito was placed on a slide under a binocular dissecting microscope (Leica LED3000, Germany), and the legs and wings were removed. The head and thorax regions were separated from the abdomen, placed in a separate drop of saline and dissected to identify potentially infective mosquitoes. Larval stages of the parasites were detected based on the morphological features and their movements (Table 1). The heads and thoraces of parasite-positive mosquitoes were stored separately for the molecular analysis of the brugian parasites, while the abdomens were stored separately for blood meal analysis at − 20 °C.

**Table 1:** Features of the brugian parasites used for the differentiation of the larval stages [49]

Features	Larval stage	
	L2	L3
Body size	Shorter and stouter	Longer and slender
Body shape	More curved or coiled	Mostly strait
Tail	Bluntly ended, not elongated	Pointed, elongated
Head	Less defined	Well defined
Body movements	Sluggish	Active

#### DNA extraction from parasite-positive mosquitoes

DNA was extracted from the heads and thoraces, and from the abdomens separately, of the parasite-positive mosquitoes using the Blood and Tissue DNA extraction kit (Qiagen, Germany) following the manufacturer’s guidelines with the following modification: DNA was eluted twice into the same tube by adding 20 μl and 15 μl of nuclease-free water, respectively, in two subsequent steps and centrifuging at 8000 rpm for 1 min after each addition.

#### Molecular confirmation of brugian infections

The *Brugia* spp.-specific *Hha1* region was amplified from the DNA extracts of the head and thorax regions of dissection-positive mosquitoes as previously described [25]. Briefly, primers 5′-GCGCATAAATTCATCAGC-3′ (forward) and 5′-GCGCAAAACTTAATTACAAAAGC-3′ (reverse) were used to amplify the 322-bp *Hha1* repeat region of *Brugia* spp. The PCR amplification was performed in a 25 μl reaction volume containing 5.0 μl of 5 × PCR buffer, 0.5 μl (0.2 mM) dNTP, 0.2 μM of each primer, 1U *Taq* DNA polymerase and 2 μl of the extracted template DNA. The PCR cycle conditions included an initial denaturation step at 94 °C for 5 min, followed by 35 cycles each of denaturation at 94 °C for 1 min, annealing at 59 °C for 1 min and extension at 72 °C for 1 min, with a final extension at 72 °C for 10 min. The products were visualized by agarose gel electrophoresis on a 1.5% agarose gel, and brugian infections were identified based on the presence of a 322-bp band. Laboratory-raised *B. malayi* and *B. pahangi* samples obtained from the Filariasis Research Reagent Resource Center (FR3) at the University of Georgia, USA, were used as positive controls, and field-collected, molecularly confirmed *Dirofilaria repens* samples from the Faculty of Medicine, University of Kelaniya, Sri Lanka, were used as the negative control for this study.

Positive PCR products from the mosquito head and thorax regions, which had concentrations sufficient for sequencing, were processed at the DNA sequencing facility at Iowa State University, USA, and Macrogen Inc., South Korea. The Basic Local Alignment and Search Tool (BLAST) on the National Centre for Biotechnology Information (NCBI) website was used to confirm the genus of the brugian parasites.

### Evidence of human blood feeding of potential brugian filariasis vectors

To determine whether these mosquitoes are involved in the transmission of *Brugia* spp. to humans, the blood meals of the infected and potentially infective mosquitoes were analyzed for human blood. DNA was extracted from the abdomens of parasite-positive mosquitoes obtained from the dissections to analyze the blood meal [26]. The 272-bp cytochrome c oxidase I (*COI*) region of the mitochondrial genome of humans was amplified using HMNF’-CTCGGCTTACTTCTCTTCC with the universal reverse primer UNVR’-AGTGGGYGRAATATTATGC. The PCR mixture was heated for 5 min at 95 °C, followed by 12 cycles of 94 °C for 30 s, 57 °C for 30 s and 72 °C for 50 s. Two additional sets of 12 cycles each followed, using decreasing annealing temperatures of 56 °C and 55 °C, respectively. A final elongation at 72 °C was performed for 5 min.

### Statistical analysis

All statistical analyses and visualizations were conducted using R 4.5.1 [27]. To assess interspecific differences in infection potential of mosquito species of brugian filariasis (Model 1) and human brugian filariasis (Model 2), we fitted two generalized linear mixed models (GLMMs) using the “glmmTMB” package with a gamma distribution and log link function using weighted indices with mosquito species included as a fixed factor in both models [28]. The approach of Kilpatric et al. was adapted with modifications to accommodate the objectives of the present study [29]. An index of vector competence was not incorporated, as relevant species-specific data were not tested experimentally.

Model 1.

The response variable was the weighted potentially infective (WPI) percentage calculated for each mosquito species as:

$${\text{WPI }} = \, P_{i} x \, A_{r} x \, 100_{e}$$

where

P_i_ = proportion of potentially infective mosquitoes of the relevant species.

A_r_ = relative abundance of the relevant species.

Model 2.

The response variable was the weighted probability of potentially infective mosquitoes with human blood (WPIH) calculated for each mosquito species at each site as:

$${\text{WPIH }} = \, P_{i} x \, A_{r} x \, P_{{{\mathrm{hb}}}} x \, 100_{e}$$

where

P_i_ = proportion of potentially infective mosquitoes of the relevant species at the relevant site.

A_r_ = relative abundance of the relevant species at the relevant site.

P_hb_ = proportion of potentially infective mosquitoes of the relevant species with human blood at the relevant site.

Model significances were evaluated using the *ANOVA* function in the “car” package [30]. Post hoc pairwise comparisons of estimated marginal means (EMMs) were performed using the “emmeans” package with Tukey’s adjustment for multiple testing [31], and species-specific weighted potential infective percentages and predicted probabilities of infectivity and presence of human blood were extracted. The predicted values with 95% confidence intervals were visualized as bar plots, and the weighted potential infectivity of mosquitoes with human blood percentages were illustrated with the “ggplot2” package [32].

A generalized linear mixed model was fitted to the “glmmTMB” package to test the effects of mosquito species, site and their interaction on both weighted indices with a Tweedie distribution with a log link function. Model significance was assessed using ANOVA. Estimated marginal means for each species-site combination was obtained using the emmeans package, and pairwise comparisons were adjusted with Tukey’s method. Non-estimable contrasts were excluded prior to post hoc analysis.

## Results

### Adult mosquito distribution and species diversity

A total of 794 mosquitoes from 15 species were captured throughout the study (Table 2). Of these, 62.7% (*n* = 498), 27.1% (*n* = 215) and 10.2% (*n* = 81) mosquitoes were found in dog-baited, gravid and window traps, respectively. The highest number of species (*n* = 14) was recorded from dog-baited traps, while seven and eight species were collected from gravid and window traps, respectively. All three types of traps captured *Mansonia annulifera, Ma. indiana, Ma. uniformis, Culex quinquefasciatus* and *Armigeres subalbatus*. In contrast, *Culex lophoceraomyia, Culex vishnui, Aedes aegypti, Aedes pipersalatus* and *Anopheles kawani* were found only in dog-baited traps, and *Culex eumelanomyia brevipalpis* was found only in window traps. The most *Culex* spp. mosquitoes were found in gravid traps. Damaged specimens were discarded, resulting in 766 mosquitoes being included in the analysis.

**Table 2:** Mosquito species collected using dog-baited (12), gravid (12) and window (12) traps operated from October 2021 to September 2022

Type of trap	Dog-baited trap	Window trap	Gravid trap
Mosquito species			
*Mansonia annulifera*	12	1	2
*Ma. indiana*	50	6	2
*Ma. uniformis*	93	5	1
*Culex gelidus*	1	0	1
*Cx. lophoceraomyia*	4	0	0
*Cx. tritaeniorhynchus*	89	0	29
*Cx. quinquefasciatus*	25	35	172
*Cx. vishnui*	2	0	0
*Cx. eumelanomyia brevipalpis*	0	3	0
*Aedes aegypti*	2	0	0
*Ae. albopictus*	40	5	0
*Ae. pipersalatus*	1	0	0
*Armigeres subalbatus*	167	23	8
*Anopheles kawani*	5	0	0
*Coquillettidia crassipes*	7	3	0
Total	498	81	215

### Detection of infected and potentially infective mosquito species

The ability of brugian parasites to develop into the infective L3 larval stage within the collected mosquito species was assessed to identify potential vectors of brugian filariasis. Mosquitoes harboring L1, L2 or L3 life stages within the head and thorax regions were identified as infected; of these, those harboring L3 parasites were identified as potentially infective because of the ability to support development to the infective stage within the vector.

**Fig. 3:**
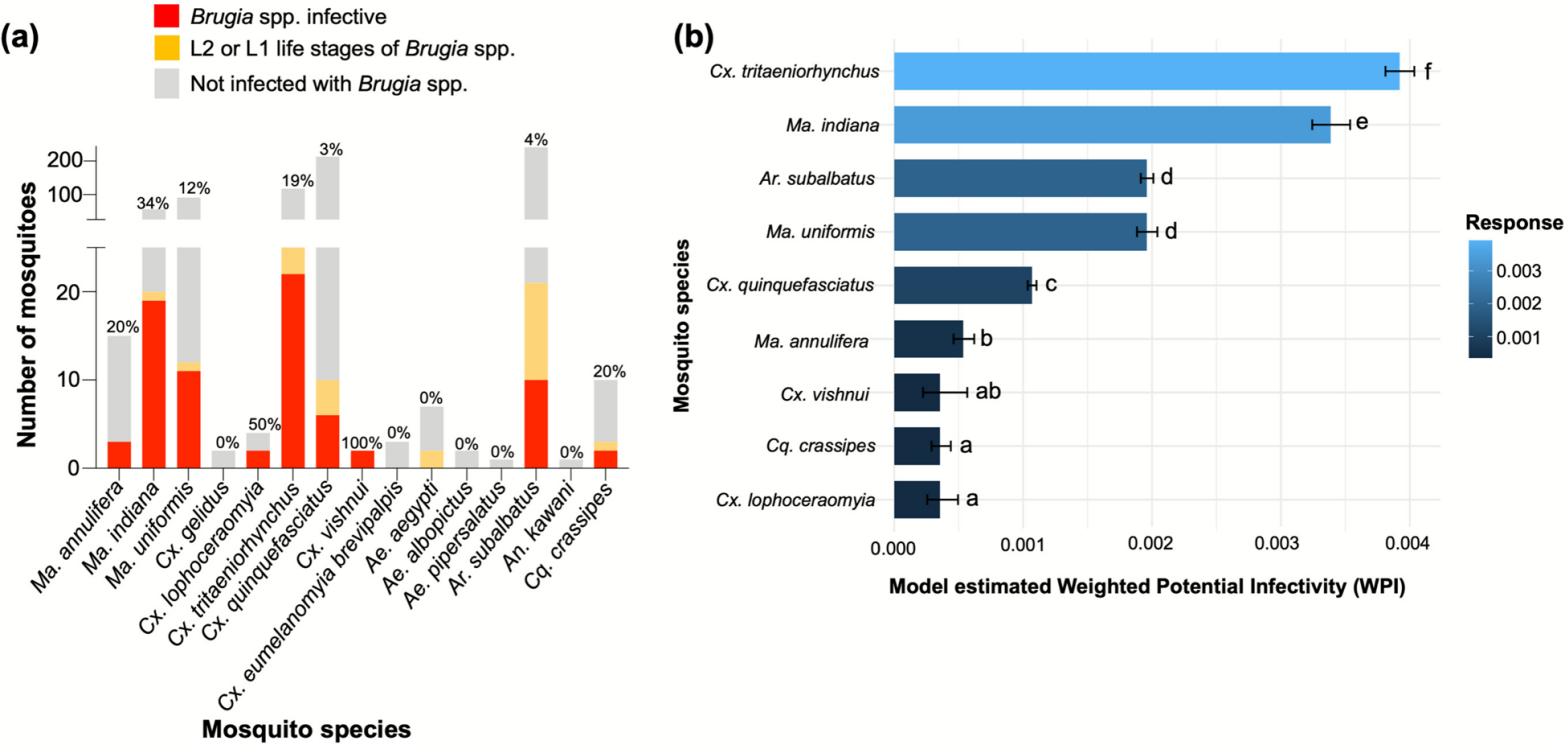
Additional mosquito species identified as potential vectors for brugian filariasis. **a** Number of mosquitoes identified with brugian infections by the presence of brugian parasites upon dissections, confirmed via PCR. The percentage of potentially infective mosquitoes of each species out of the total collection is represented on the bars. **b** Predicted weighted potential infectivity as a percentage of the total potentially infective mosquito species. The error bars represent SE. The compact letters indicate statistically significant differences among species based on pairwise comparisons (*p* < 0.05)

Of the 15 species of mosquitoes caught, *Ma. annulifera, Ma, indiana, Ma. uniformis, Cx. lophoceraomyia, Culex tritaeniorhynchus, Cx. quinquefasciatus, Cx. vishnui, Ae. aegypti, Ar. subalbatus* and *Coquillettidia crassipes* species (*n* = 10) were infected, and all but *Ae. aegypti* were potentially infective (Fig. 3), suggesting their potential to serve as vectors of brugian filariasis in Sri Lanka.

The GLMM showed significant differences in potential infectivity probability among mosquito species (χ^2^ = 24,377, *df* = 9, *p* < 0.001). Weighted infective capability varied significantly among mosquito species. *Culex tritaeniorhynchus* (*n* = 22, 19%, mean ± SD = 0.00384 ± 0.00004), followed by *Ma. indiana* (*n* = 19, 34%, mean ± SD = 0.00332 ± 0.00005), were identified as the species with the highest predicted weighted potential infectivity percentages (Additional file 1). Compared with other potentially infective species, *Ar. subalbatus* harbored the most L1- and L2-stage brugian parasites (*n* = 11, 4.6%) in addition to a considerable number of L3 stage parasites (*n* = 10, 4%). This may reflect a transitional period in which *Ar. subalbatus* is being manipulated by the brugian parasite to support its development to the infective stage. *Mansonia annulifera*, *Cx. lophoceraomyia* and *Cx. vishnui* exhibited comparatively lower predicted weighted potential infectivity percentages. Mosquito species where no brugian parasites were detected were eliminated from further analysis.

### Evidence of human blood feeding of potential brugian filariasis vectors

**Fig. 4:**
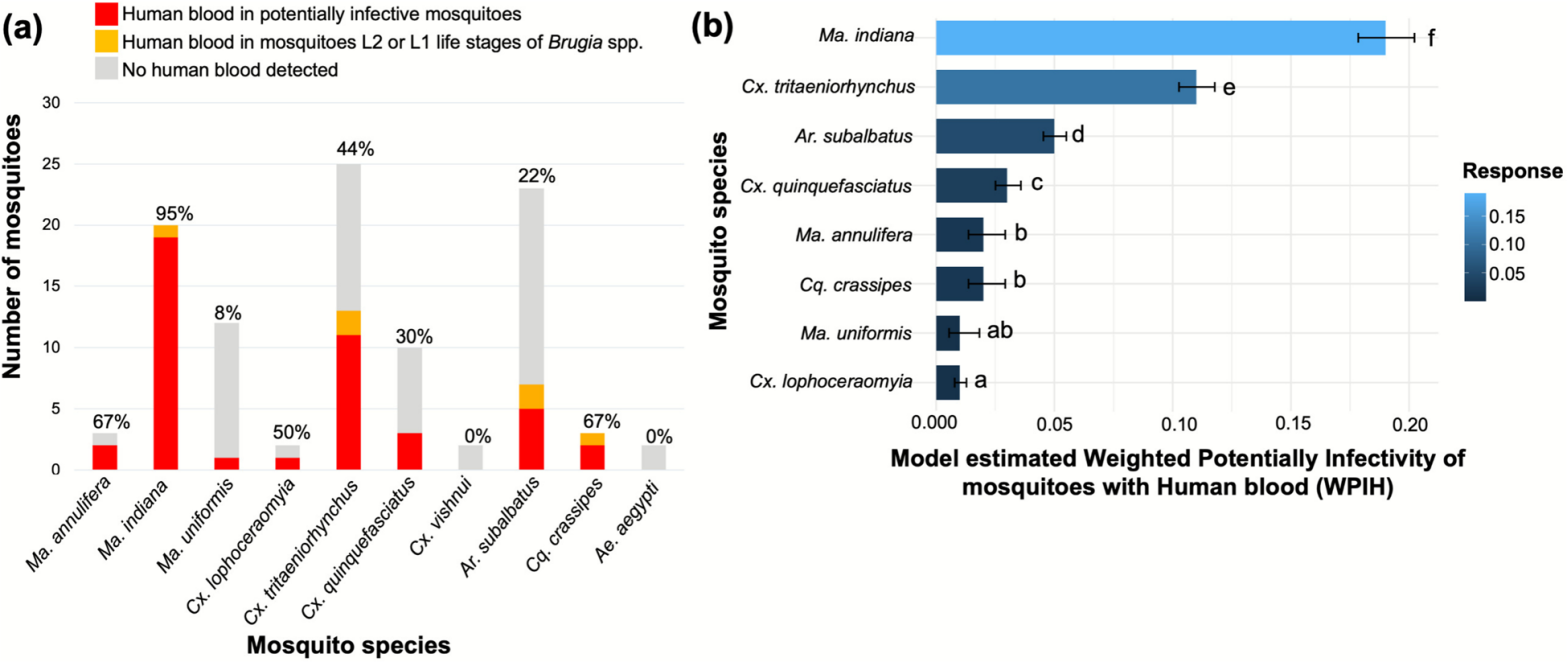
Potential brugian vectors showed evidence of human blood feeding. **a** Presence of human blood in infected mosquitoes. Of the infected mosquitoes of each species, potentially infective mosquitoes detected with human blood are represented as a percentage on the bars. **b** Predicted weighted potential infectivity as a percentage of the total potentially infective mosquito species with human blood. The error bars represent SE. The compact letters indicate statistically significant differences among species based on pairwise comparisons (*p* < 0.05)

To examine the potential involvement of mosquito species capable of transmitting human brugian filariasis, they were tested for traces of human DNA. Human blood was detected in all the potentially infective species except *Cx. vishnui*, suggesting their potential in transmission of human brugian filariasis (Fig. 4)*.* Interestingly, the highest potential infectivity percentage with human blood was found in *Ma. indiana* (*n*_infected_ = 20, *n*_infective with human DNA_ = 19, mean ± SD = 0.00338 ± 0.00005) followed by *Cx. tritaeniorhynchus* (*n*_infected_ = 13, *n*_infective with human DNA_ = 11, mean ± SD = 0.00196 ± 0.00002) (Additional file 2). As the two species with the highest percentage of potentially infective mosquitoes with human DNA, *Ma. indiana* and *Cx. tritaeniorhynchus* could potentially serve as vectors of human brugian filariasis in Sri Lanka in addition to the already established *Ma. annulifera* and *Ma. uniformis* vectors.

### Site-based risk analysis of brugian filariasis

**Fig. 5:**
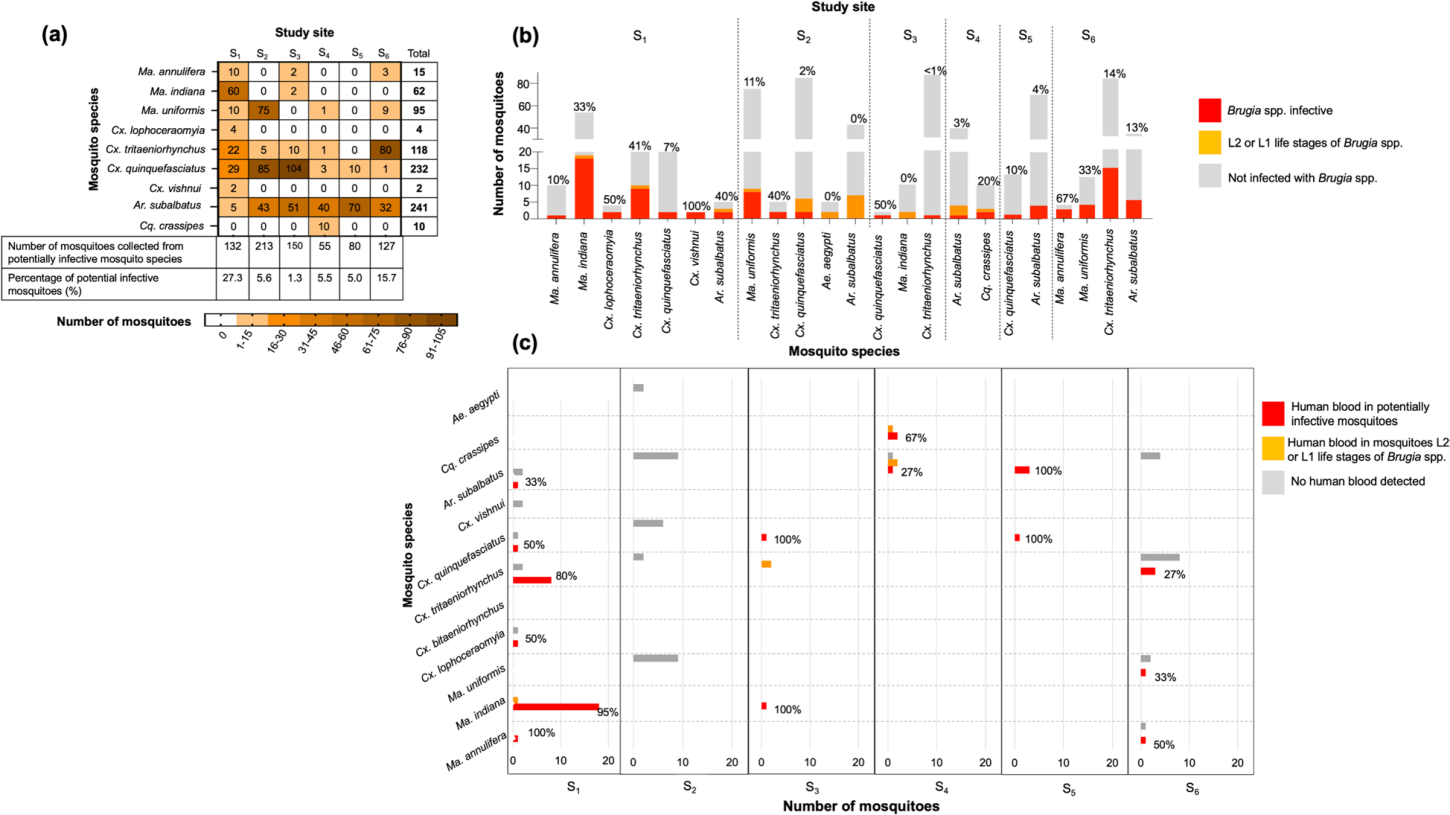
Risk analysis between the study sites. Puruduwella-Puttalam District (S_1_), Maggona-Kalutara District (S_2_), Wattala-Gampaha District (S_3_), Induruwa-Galle District (S_4_), Boralesgamuwa-Colombo District (S_5_) and Mahawewa-Puttalam District (S_6_). **a** Heat map of the distribution of the mosquitoe species in which brugian parasites were detected.** b** Site-wise species distribution of potentially infective mosquitoes. Potentially infective mosquitoes of each species are represented as a percentage of the dissected mosquitoes on the bars. **c** Distribution of infected mosquito species with human blood. Potentially infective mosquitoes of each species are represented as a percentage of infected mosquitoes on the bars

Mosquito species in which brugian parasites were detected (Fig. 3) were further analyzed to assess their spatial distribution. A site-wise analysis of the abundance of *Brugia*-positive mosquito species and total percent infectivity was carried out (Fig. 5a). Furthermore, to identify the dominant species and evaluate the risk of brugian filariasis at each site, the percentages of infected and infective mosquitoes (Fig. 5b) were calculated. To investigate the risk of transmission of human brugian filariasis at each site, infected and potentially infective mosquito species were assessed for human DNA across the six sites (Fig. 5c).

The total abundance of *Brugia*-positive mosquito species was highest at S_2_ (*n* = 213) and S_3_ (*n* = 150), closely followed by S_1_ (*n* = 132) and S_6_ (*n* = 127), and lowest at S_4_ (*n* = 55), with the highest percentage of potentially infective mosquitoes at S_1_ (*n* = 36, 27.3%) and S_6_ (*n* = 20, 15.7%). Of the nine species identified as potentially infective, S_1_ was the most species-rich site (*n* = 8), followed by S_6_ (*n* = 5). Interestingly, all the species at S_1_ and S_6_ were potentially infective, with *Ma. indiana* having the most potentially infective mosquitoes at S_1_ (*n* = 18, 33%), followed by *Cx. tritaeniorhynchus* (*n* = 29, 41%). The GLMM results showed significant effects of both mosquito species and site on the weighted potential infectivity index. The effects of species (χ^2^ = 586.72, df = 8, *p* < 0.001), site (χ^2^ = 689.32, df = 5, *p* < 0.001) and their interaction (χ^2^ = 700.13, df = 15, *p* < 0.001) were all significant. Regarding potential infectivity, *Ma. indiana* and *Cx. tritaeniorhynchus* from S_1_ and *Cx. tritaeniorhynchus* from S_6_ showed the highest weighted values for potentially infective mosquitoes, where differences were significant (*p* < 0.001). In contrast, *Ar. subalbatus, Ma uniformis* and *Ma. annulifera* generally showed lower infectivity across most sites.

Human blood was detected in all potentially infective species at S_1_ except *Cx. vishnui* with the highest proportion detected in *Ma. indiana* (*n* = 18, 95%), followed by *Cx. tritaeniorhynchus* (*n* = 8, 80%,). Significant differences were observed among mosquito species (χ^2^ = 599.59, df = 8, *p* < 0.001), among sites (χ^2^ = 2000.02, df = 5, *p* < 0.001) and for the species-site interaction (χ^2^ = 987.76, df = 15, *p* < 0.001) when considering the weighted number of mosquitoes with human blood. This suggests that the proportion of potential vectors with human blood varied substantially among mosquito species and across sites, with significant spatial heterogeneity in species-specific responses.

Pairwise comparisons showed significant variation in both the weighted potential infectivity of mosquitoes with human blood and across sampling sites. Accordingly, *Ma. indiana* (*p* < 0.001) followed by *Cx. tritaeniorhynchus* (*p* < 0.001) from S_1_ can be identified as potential vectors for human brugian filariasis. Human blood was detected in three of the four potentially infective species at S_6_ (*n* = 4, 20%). However, the number of mosquitoes of individual species is insufficient to identify potential vectors at S_6_.

Although the total abundance was highest at S_2_ and S_3_, the percentage of infectivity was low (S_2_ = 5.6%, S_3_ = 1.3%), with no human blood being detected in infective mosquitoes at S_2_. These data collectively suggest that S_1_ is at the highest risk of transmission of human brugian filariasis, followed by S_6_, with *Ma. indiana* and *Cx. tritaeniorhynchus* potentially serving as additional vectors at S_1_.

## Discussion

Brugian filariasis has re-emerged in Sri Lanka after 4 decades of quiescence, with an increase in disease incidence in 2023. The absence of a definitive cure for LF in its advanced stages underscores the critical importance of prevention strategies, particularly through effective vector control and MDA. This study aimed to identify potential vectors of brugian filariasis in Sri Lanka to support targeted vector control measures.

Here, we present evidence of infection and development of brugian parasites to the infective L3 larval stage in field-caught *Ma. annulifera, Ma. indiana, Ma. uniformis, Cx. lophoceraomyia, Cx. tritaeniorhynchus, Cx. quinquefasciatus, Cx. vishnui, Ar. subalbatus* and *Cq. crassipes* in Sri Lanka. To date, only *Ma. annulifera* and *Ma. uniformis* have been reported as potential vectors for brugian filariasis in Sri Lanka [17, 21, 33]. The presence of infective brugian parasites in field-caught *Ma. indiana, Cx. lophoceraomyia, Cx. tritaeniorhynchus, Cx. quinquefasciatus, Cx. vishnui, Ar. subalbatus* and *Cq. crassipes* species have not been reported in Sri Lanka [17, 21, 33] and *Cx. lophoceraomyia, Cx. quinquefasciatus* and *Cx. vishnui* in the world [34–41]. To our knowledge, this is the first study in Sri Lanka to systematically collect mosquitoes using multiple collection methods to ensure maximum species diversity in the collection, enabling the detection of a broader range of potentially infective vector species.

This study reports an unusually higher number of *Ma. indiana* mosquitoes compared with previous studies in Sri Lanka. Two studies, which analyzed 82 and 1060 mosquitoes respectively, reported three and two *Ma. indiana* mosquitoes, none of which were infected with the brugian parasite [17, 33], and no *Ma. indiana* mosquitoes were reported in the survey conducted over 6 consecutive months, in which nearly 7000 mosquitoes were analyzed [21]. Vector studies on brugian filariasis are scarce in Sri Lanka, where only the above three studies have been conducted, and all three were carried out in the Gampaha District. Our results of the study site in Gampaha align with the results of the above studies where, of the 150 mosquitoes collected, only two *Ma. indiana* mosquitoes were present; however, both were infected with brugian parasites. The high abundance of *Ma. indiana* observed in this study may be attributable to extending the study beyond the Gampaha District, allowing for the identification of geographical pockets of certain species. Furthermore, seasonal climatic factors and favorable environmental conditions at the time of sampling could have influenced the presence of brugian parasites within these mosquitoes; however, these parameters were beyond the scope of the present study and were not formally evaluated. However, *Ma. indiana* has been reported in abundance in filariasis-endemic areas in other countries and has been reported as a potential vector for filariasis in Thailand, Malaysia and Indonesia [35–37]. Studies have shown its capacity to transmit nocturnally sub-periodic *B. malayi* parasites [36], supporting its role as a potential vector for brugian filariasis. *Culex tritaeniorhynchus* and *Ae. albopictus* have been reported as vectors of *B. malayi* in Indonesia [37], while *Ar. subalbatus* has been identified as a vector for zoonotic *B. pahangi* in Thailand [38] and Malaysia [38, 39] and *Cq. crassipes* as a vector for *B. malayi* in Malaysia [36].

Interestingly, the presence of infective L3 *Brugia* spp. larvae in field-caught *Cx. lophoceraomyia, Cx. quinquefasciatus* and *Cx. vishnui* has not been reported elsewhere thus far. In 1995, Bangs et al. reported the susceptibility of *Culex tarsalis* and *Culex erythrothorax* to sub-periodic *B. malayi* through laboratory experiments, thereby providing evidence for the ability of the genus *Culex* to act as a vector for brugian filariasis [44]. Interestingly, some laboratory experiments demonstrated the complete refractoriness of *Culex sitiens* to sub-periodic *B. malayi* [38], and the mid-gut was shown to act as a barrier against brugian filarial parasites in *Cx. pipiens pipiens* [45]. However, a study done by Erickson et al. reported the presence of filarial DNA within the head region of *Cx. pipiens* [46]. Ughasi et al. reported the possibility that mosquito species previously considered non-vectors could act as vectors of filariasis parasites over generations [47]. This may reflect a parasite with genetic modifications, which requires further investigation. Detection of human blood in potentially infective *Ma. annulifera, Ma. indiana, Ma. uniformis, Cx. lophoceraomyia, Cx. tritaeniorhynchus, Cx. quinquefasciatus, Ar. subalbatus* and *Cq. crassipes* suggests a potential role of these species in the transmission of human brugian filariasis.

The S_1_ site was identified as the area with the highest risk regarding human brugian filariasis at the time of the study, when considering abundance, species richness of *Brugia*-positive mosquitoes, percentages of potentially infective mosquitoes and the proportion of them with traces of human blood. Incidentally, the S_1_ site is in the Puttalam District, which had the highest disease incidence (53 cases) from 2001 to 2021, as identified through human blood TBS observations by the AFC. Interestingly, *Ma. indiana* was by far the most prevalent and potentially infective mosquito species at study site S_1_, closely followed by *Cx. tritaeniorhynchus*. With a high abundance, a high percentage of infectivity and the presence of human blood within them, *Ma. indiana* and *Cx. tritaeniorhynchus* could potentially contribute to the transmission of human brugian filariasis in these areas. Their absence in S_4_ and S_5_ and low infectivity percentages of infectivity in S_2_ and S_3_ could have been reasons for the low disease incidence (S_2_ = 32, S_3_ = 23, S_4_ = 10, S_5_ = 6) in these areas at the time of the study.

The presence of infective mosquitoes and a high abundance of potential vector species at S_2_ suggests the potential for transmission of brugian filariasis. However, the low percentage of infectivity of these mosquitoes and the absence of human blood within them could be suggestive of a zoonotic brugian filariasis in the area or a recent introduction of brugian filariasis to the area and, therefore, the initial stages of transmission at the time of the study. Although human brugian filariasis has not yet been detected within the S_2_ study site, the presence of the potential vector species indicates the risk of developing new cases of human brugian filariasis within this site.

In the current study, the entire mosquito catch was analyzed for brugian infections by dissection. However, currently, in the national vector surveillance program of the AFC, only *Ma. annulifera* and *Ma. uniformis* (previously known vector for brugian filariasis) mosquitoes are dissected for brugian filariasis infections, since *Ma. indiana* has not been found so far. Mosquitoes are dissected for brugian filariasis infections and pooled for PCR to detect brugian infections. The identification of new potential vectors suggests the importance of extending the xenomonitoring efforts to other species for a comprehensive analysis of the situation.

However, the current survey is not ideal for comparing the geographical distribution of vectors, as seasonal variation also plays an important role in determining mosquito populations. The survey was not conducted in comparable climatic seasons; mosquitoes were collected during the inter-monsoon season at S_1_ and during the monsoon season at S_2_-S_6_. Therefore, conducting mosquito collections in comparable seasons would help reveal the true geographical and seasonal patterns of potential vector distribution across the sites. Furthermore, the present study was intended to provide site-specific baseline entomological evidence following LF elimination in Sri Lanka and was not designed to support extrapolation beyond the study area. Nevertheless, it constitutes the first national-level effort to evaluate all mosquito species collected for potential vector status.

In the present study, mosquitoes carrying L3 larvae were identified as potentially infective because of the ability of those species to support the development of the parasites to the infective stage. However, Gleave et al. demonstrated the inability of certain mosquitoes to transmit the disease [48]. Therefore, the mosquito species identified in this study as potential vectors of brugian filariasis should undergo further validation through experimental vector competence studies. Such follow-up research is essential to accurately determine the role of these mosquito species in the transmission of brugian filariasis and to guide targeted vector control strategies. Data generated by the current study could be used to refine prevention and control strategies by developing more tailored vector control measures in Sri Lanka and other brugian filariasis-endemic countries. Such measures are crucial in preventing the potential resurgence of the disease.

The identification of new potential vector species for brugian filariasis, including several not previously reported in Sri Lanka or globally, reveals significant shifts in the local vector ecology and transmission dynamics. Incorporating multidisciplinary surveillance combining entomological, ecological and molecular data will be essential to detect emerging vector species, assess their public health significance and design context-specific control strategies. Such integrated efforts are critical for achieving sustainable elimination of human brugian filariasis.

## Conclusions

This study demonstrates that brugian parasites are present in a wide range of mosquito species in post-elimination Sri Lanka, with seven species across four genera capable of supporting parasite development to the infective stage. The identification of *Ma. indiana* as a highly competent species in Sri Lanka represents a novel finding with significant epidemiological implications. These findings provide essential baseline data for evidence-based vector control strategies in Sri Lanka’s post-elimination LF program. Nonetheless, gaps in knowledge remain regarding the natural transmission capacity, seasonal variations and vector-parasite interactions under field conditions. The diversity of competent species suggests complex transmission dynamics that require comprehensive, adaptable surveillance strategies to prevent the re-establishment of endemic transmission. Future research priorities include experimentally validating vector competence, conducting seasonal surveillance and assessing natural transmission capacities, especially with *Ma. indiana* and *Cx. tritaeniorhynchus*, for the transmission of brugian filariasis.

## Supplementary Information

Additional file 1.

Additional file 2.

## Data Availability

Data supporting the main conclusions of this study are included in the manuscript.

## Abbreviations

LF: Lymphatic filariasis
MDA: Mass drug administration
AFC: Anti-filariasis campaign
TBS: Thick blood smear
ITS2: Internal transcribed spacer region 2
BLAST: Basic Local Alignment and Search Tool
NCBI: National Centre for Biotechnology Information
GLMM: Generalized linear mixed models
EMM: Estimated marginal mean
WPI: Weighted potential infectivity
WPIH: Weighted potential infectivity percentage of mosquitoes with human blood
