# Supplementary material for: Post elimination of lymphatic filariasis: a situation analysis of brugian filariasis and vector potentialities within the filarial transmission belt in Sri Lanka
Source: Parasit Vectors. 2026 Mar 6;19:108. doi: 10.1186/s13071-026-07264-w (PMC12973600; doi:10.1186/s13071-026-07264-w)
Supplement: Supplementary file 2 — Additional file 2. [file 13071_2026_7264_MOESM2_ESM.docx]

| Species | Estimate | Std_Error | Lower_CI | Upper_CI |
| --- | --- | --- | --- | --- |
| *Cx. lophoceraomyia* | 0.0003565 | 4.23102642971887E-05 | 0.00025675 | 0.000495 |
| *Cq. crassipes* | 0.00035651 | 2.67597372183857E-05 | 0.00028968 | 0.00043875 |
| *Cx. vishnui* | 0.00035651 | 5.98371200880318E-05 | 0.00022412 | 0.0005671 |
| *Ma. annulifera* | 0.00053476 | 2.88682628786142E-05 | 0.0004606 | 0.00062086 |
| *Cx. quinquefasciatus* | 0.00106952 | 1.23338728665616E-05 | 0.00103595 | 0.00110418 |
| *Ma. uniformis* | 0.00196078 | 2.86173543208756E-05 | 0.00188321 | 0.00204154 |
| *Ar. subalbatus* | 0.00196078 | 1.76215933763934E-05 | 0.00191265 | 0.00201013 |
| *Ma. indiana* | 0.0033868 | 5.31051347996273E-05 | 0.00324308 | 0.0035369 |
| *Cx. tritaeniorhynchus* | 0.00392156 | 4.04606801047037E-05 | 0.00381124 | 0.00403507 |

**Table 3:** Model-estimated Weighted Potential Infectivity of mosquitoes with human blood (WPIH) corresponding to each mosquito species.

**Table 4:** Raw Weighted Potential Infectivity percentage of mosquitoes with human blood (WPIH) corresponding to each mosquito species.

| Mosquito species | Number of mosquitoes found in each species | Number of totally infected mosquitoes | Number of potentially infective mosquitoes with human blood | Relative abundance of the relevant species (P_i_) | Proportion of potentially infective mosquitoes of the relevant species (A_r_) | Relative abundance (Phb) | Weighted Potentially Infective Percentage of mosquitoes with Human blood (WPIH) |
| --- | --- | --- | --- | --- | --- | --- | --- |
| *Ma. annulifera* | 15 | 3 | 2 | 0.20 | 0.02 | 0.67 | 0.26 |
| *Ma. indiana* | 56 | 19 | 19 | 0.34 | 0.07 | 1.00 | 2.48 |
| *Ma. uniformis* | 91 | 11 | 1 | 0.12 | 0.12 | 0.09 | 0.13 |
| *Cx. lopoceraomyia* | 4 | 2 | 1 | 0.50 | 0.01 | 0.50 | 0.13 |
| *Cx. tritaeniorhynchus* | 118 | 22 | 11 | 0.19 | 0.15 | 0.50 | 1.44 |
| *Cx. quinquefasciatus* | 213 | 6 | 3 | 0.03 | 0.28 | 0.50 | 0.39 |
| *Cx. vishnui* | 2 | 2 | 0 | 1.00 | 0.00 | 0.00 | 0.00 |
| *Ar. subalbatus* | 241 | 10 | 5 | 0.04 | 0.31 | 0.50 | 0.65 |
| *Cq. crassipes* | 10 | 2 | 2 | 0.20 | 0.01 | 1.00 | 0.26 |
